# NH_2_^+^ implantations induced superior hemocompatibility of carbon nanotubes

**DOI:** 10.1186/1556-276X-8-205

**Published:** 2013-05-01

**Authors:** Meixian Guo, Dejun Li, Mengli Zhao, Yiteng Zhang, Xiangyun Deng, Dongsheng Geng, Ruying Li, Xueliang Sun, Hanqing Gu, Rongxin Wan

**Affiliations:** 1 College of Physics and Electronic Information Science, Tianjin Normal University, Tianjin, 300387, China; 2 Department of Mechanical & Materials Engineering, University of Western Ontario, London, ON, Canada; 3 Tianjin Institute of Urological Surgery, Tianjin Medical University, Tianjin 300070, China; 4 Ninth People’s Hospital, Shanghai Jiao Tong University, School of Medicine, Shanghai 200011, China

**Keywords:** Multiwalled carbon nanotubes, Ion implantation, Hemocompatibility, Functional group

## Abstract

NH_2_^+^ implantation was performed on multiwalled carbon nanotubes (MWCNTs) prepared by chemical vapor deposition. The hemocompatibility of MWCNTs and NH_2_^+^-implanted MWCNTs was evaluated based on *in vitro* hemolysis, platelet adhesion, and kinetic-clotting tests. Compared with MWCNTs, NH_2_^+^-implanted MWCNTs displayed more perfect platelets and red blood cells in morphology, lower platelet adhesion rate, lower hemolytic rate, and longer kinetic blood-clotting time. NH_2_^+^-implanted MWCNTs with higher fluency of 1 × 10^16^ ions/cm^2^ led to the best thromboresistance, hence desired hemocompatibility. Fourier transfer infrared and X-ray photoelectron spectroscopy analyses showed that NH_2_^+^ implantation caused the cleavage of some pendants and the formation of some new N-containing functional groups. These results were responsible for the enhanced hemocompatibility of NH_2_^+^-implanted MWCNTs.

## Background

Antithrombogenic biomaterial is being extensively studied in order to fabricate artificial organs and biomedical materials in contact with blood. A significant goal for the application of antithrombogenic biomaterial is to prevent thrombus formation on material surface. Thrombus formation involves a process with multiple steps, including plasma protein adsorption, platelet adhesion and aggregation, and finally, the activation of clotting factor. The properties of the surface such as hydrophobicity/hydrophilicity, surface charge, and roughness of biomaterials strongly influence platelet adhesion, activation, and thrombus formation when the surface is in contact with blood [1].

The unusual mechanical properties of carbon nanotubes (CNTs) such as high hardness, low coefficient of friction, and high wear and corrosion resistance render them an ideal class of reinforcement for multiple biomedical applications including tissue engineering, biomedicine, biomaterials, (bio) sensors, catalysts, and so on [2-12]. However, the hydrophobicity and inertness of CNTs frequently hinder their biomedical application. So, surface modification of CNTs is very important to minimize the adverse interaction and improve the biocompatibility in clinical applications.

According to previous works, many results on surface modification of polymers induced by pure individual chemical element ion implantation to control their biocompatibility have been reported [13-22]. Ion implantation is one of the most powerful techniques for the surface modification of solids. It has been applied to the surface modification of polymers in order to control conductive, mechanical, physical, and chemical properties [23-27]. This technique has many advantages in application. In addition to the technological simplicity and cleanliness, it modifies only the surface characteristics without affecting the bulk properties. Therefore, if a biomaterial with the desired bulk properties does not exhibit the appropriate biocompatibility, its surface can be modified by this technique [28].

In this work, multiwalled carbon nanotubes (MWCNTs) prepared by chemical vapor deposition (CVD) were implanted by NH_2_ ions. We chose NH_2_^+^ as the implanted ions for the reason that the -NH_2_ and -NH amidogen radicals as two functional groups commonly in organic molecules are vital to the activities of living tissues; it is natural to consider grafting amidogen radicals to the surface of MWCNTs to activate the surface and further improve their biocompatibility. So, NH_2_^+^-implanted MWCNTs (NH_2_/MWCNTs) are supposed to be excellent candidates for applications as biocompatible materials in biomedical implants. So far, however, few reports that NH_2_^+^ implantation is used to improve biocompatibility, especially hemocompatibility of MWCNTs, can be found. Our purpose, in this work, is to introduce N-containing functional groups to the surface of MWCNTs by NH_2_^+^ implantation and insight into the influence of implanted fluency on its hemocompatibility.

## Methods

### Preparation and characterization of MWCNTs and NH_2_/MWCNTs

The syntheses of MWCNTs were carried out utilizing a CVD system at 800°C to 850°C with argon and ethylene gas flowing rates of 250 and 100 sccm, respectively. Then, MWCNTs were dissolved in deionized water with ultrasonic dispersion for 5 min. After centrifugation of 10 min at the speed of 1,000 rpm in a tabletop microcentrifuge, the upper supernatant-containing MWCNTs were directly sprayed onto the SiO_2_ substrates using airbrush pistol at 100°C to prepare pristine MWCNT samples.

The implantation was carried out using a BNU-400 keV implanter (Beijing Normal University, Beijing, China). The NH_2_^+^ generated from gaseous NH_3_ was identified by mass spectrometry. The collected NH_2_^+^ was then accelerated in a high voltage onto the MWCNT samples. During implantation, the NH_2_^+^ energy was 30 keV, the beam current density was controlled under 4 μA/cm^2^. The fluencies of 5.0 × 10^14^ and 1.0 × 10^16^ ions/cm^2^ were chosen for a comparison.

The chemical composition of the samples was determined by Fourier transform infrared spectroscopy (FTIR, MAGNA-560, Nicolit, USA). X ray photoelectron spectroscopy (XPS, PHI5000, ULVAC-PHI, Inc., Chigasaki City, Japan) was employed to determine the chemical bonding states and content bonds. Analysis was performed using a versa probe system. Contact angle measurements were performed on the samples’ surface using a CAM 200 optical contact-angle inclinometer (Nunc, Finland). The results were the mean of ten measurements taken on different regions of the surface. To avoid cross-contamination of liquids, a dedicated microsyringe was used for each liquid. The morphology of the samples was examined with a field emission scanning electron microscope (FESEM, 18SI, FEI, Czech Republic) operated at 10 kV and transmission electron microscopy (TEM, G^2^F20, FEI, USA).

### Platelet adhesion test

The *in vitro* hemocompatibility of the samples was evaluated by the platelet adhesion test. The platelet-rich plasma (PRP) was prepared by centrifuging rabbit whole blood which contained 2 wt.% potassium oxalate solution (blood:potassium oxalate = 9:1) at 1,000 rpm for 15 min. Methylsilicone oil has excellent anticoagulant activity, but quartz causes coagulation, so we chose quartz glasses with and without methylsilicone oil as reference groups. The samples as well as reference groups were placed in 24-well microplates; then, 0.7 ml PRP was injected into each well and incubated at 37°C for 30 min, the weakly adhered platelets were rinsed by phosphate buffer solution. The platelet adhesion rate of a material can be calculated as follows: $\mathrm{Platelet}\;\mathrm{adhesion}\;\mathrm{rate}\;\left(\%\right)=\frac{A-B}{A}\times 100\%$, where *A* is the total number of platelets, and *B* is the number of platelets remaining in the blood after the platelet adhesion test.

### Hemolysis test

Hemolysis can determine the volume of hemoglobin released from red blood cells (RBCs) adhered on the surfaces of the samples. Anticoagulated blood was prepared from 20 ml healthy rabbit blood plus 1 ml 2 wt.% potassium oxalate. Anticoagulated blood solution was obtained using anticoagulated blood mixed with normal saline (NS) at 1:1 volume ratio. MWCNT and NH_2_/MWCNT samples were placed in each Erlenmeyer flask with 5 ml normal saline. The same numbers of Erlenmeyer flasks with either 5 ml NS or distilled water were used as negative and positive control groups, respectively. After heating in water bath at ±37°C for 30 min, 0.7 ml anticoagulated blood solution was injected into the flasks of each group, then shaken and heated at ±37°C for 60 min. The supernatant was removed after centrifugation for 15 min at 1,000 rpm. The optical density (OD) at 545 nm was measured with a spectrophotometer. OD_545nm_ values were related to the concentration of free hemoglobin in supernatant due to broken red blood cells. The hemolytic rate is calculated by the formula: $\mathrm{Hemolytic}\;\mathrm{rate}\;\left(\%\right)=\frac{A-B}{C-B}\times 100\%$, where *A*, *B*, and *C* are the absorbance values of the samples, negative control group (physiological salt water), and positive control group (H_2_O).

### Kinetic blood-clotting time assay

Kinetic blood-clotting time was tested by the kinetic method. Blood (0.2 ml) from a healthy adult rabbit was immediately dropped onto the surface of all samples. After 5 min, the samples were transferred into a beaker which contained 50 ml of distilled water. The red blood cells which had not been trapped in a thrombus were hemolytic, and the free hemoglobin was dispersed in the solution. The concentration of free hemoglobin in the solution was colorimetrically measured at 540 nm with a spectrophotometer. The optical density at 540 nm of the solution vs. time was plotted. In general, the OD_540 nm_ value decreases with the blood-clotting time.

## Results and discussion

**Figure 1:**
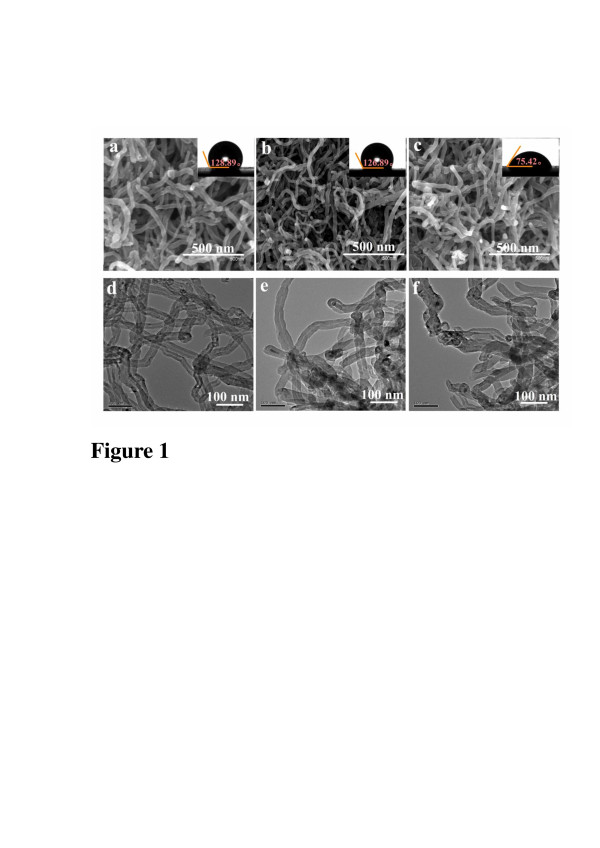
**SEM and TEM images with contact angle images of MWCNTs and NH2/MWCNTs.** SEM images of (**a**) pristine MWCNTs, (**b**) NH^2^/MWCNTs with 5 × 10^14^ ions/cm^2^, (**c**) NH^2^/MWCNTs with 1 × 10^16^ ions/cm^2^. TEM images of (**d**) pristine MWCNTs, (**e**) NH2/MWCNTs with 5 × 10^14^ ions/cm^2^, (**f**) NH^2^/MWCNTs with 1 × 10^16^ ions/cm^2^. The insets are their contact angle images, respectively.

SEM and TEM images of MWCNTs and NH_2_/MWCNTs are shown in Figure 1. It is obvious that frizzy MWCNTs entangle together with long tubes and closed pipe ports (Figure 1a,d). In contrast, NH_2_/MWCNTs in the formation of small bundles on the surface are broken, and most of the pipe ports are open (Figure 1b,c,e,f). According to the previous study [29], we believe that the implanted MWCNTs form active centers on the surface, which may increase the catalytic activity of the blood components.

To investigate the enhancement mechanism, the calculated results of the surface tension between the samples and water are shown in the insets of Figure 1. These contact angle values provide an objective explanation on the wettability of the samples which is relative to the adhesion behavior of the platelets. It is clear that the contact angle of water and surface tension of NH_2_/MWCNTs are relatively low, indicating that NH_2_^+^ implantation induces an increase in the hydrophilicity of MWCNTs.

**Figure 2:**
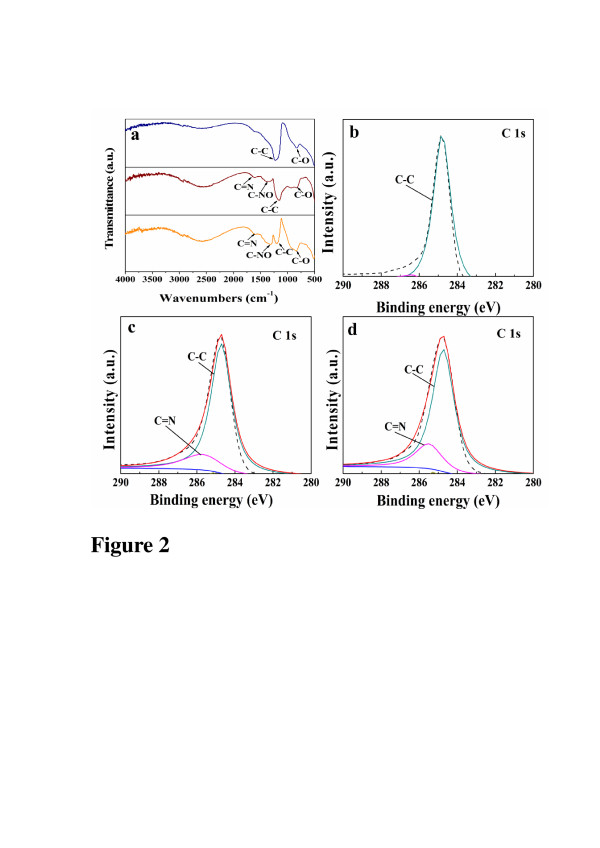
**Transmission spectra of MWCNTs and NH_2_/MWCNTs.** (**a**) FTIR spectra of pristine MWCNTs and NH_2_/MWCNTs with 5 × 10^14^ and 1 × 10^16^ ions/cm^2^. C1s XPS spectra obtained from (**b**) pristine MWCNTs, (**c**) NH_2_/MWCNTs with 5 × 10^14^ ions/cm^2^, and (**d**) NH_2_/MWCNTs with 1 × 10^16^ ions/cm^2^.

In order to analyze the changes of the functional groups caused by the NH_2_^+^ implantation, FTIR analysis is peformed. Figure 2a shows the transmission spectra of the pristine MWCNTs and NH_2_/MWCNTs with fluencies of 5 × 10^14^ and 1 × 10^16^ ions/cm^2^. Among many peaks, the peak at 1,200.11 cm^−1^ corresponds to C-C stretching vibration, while the peak at 836.69 cm^−1^ corresponds to C-O stretching vibration. NH_2_^+^ implantation produces new peaks at 1,319.56 cm^−1^ corresponding to C-NO stretching vibration and at C=N stretching vibration at 1,601.69 cm^−1^. This result proves the decomposition of some chemical bonds and formation of new N-containing functional groups.

High-resolution C1s peaks of the samples presented in Figure 2b,c,d show more detailed chemical modification after NH_2_^+^ implantation. Compared with the corresponding peak obtained from the pristine sample, the high-resolution C1s peak of NH_2_/MWCNTs appears as a new C=N bond, and meanwhile, the C-C bond declines, indicating that some pristine C-C bonds are broken by ion implantation to reconstruct new bonds with N. What is more, the spectrum of the implanted sample with fluency of 1 × 10^16^ ions/cm^2^ displays higher intensity of C=N bond at 285.5 eV as compared with the spectrum of the implanted sample with 5 × 10^14^ ions/cm^2^, which proves that higher content of N element can be obtained with the higher implanted fluency.

**Figure 3:**
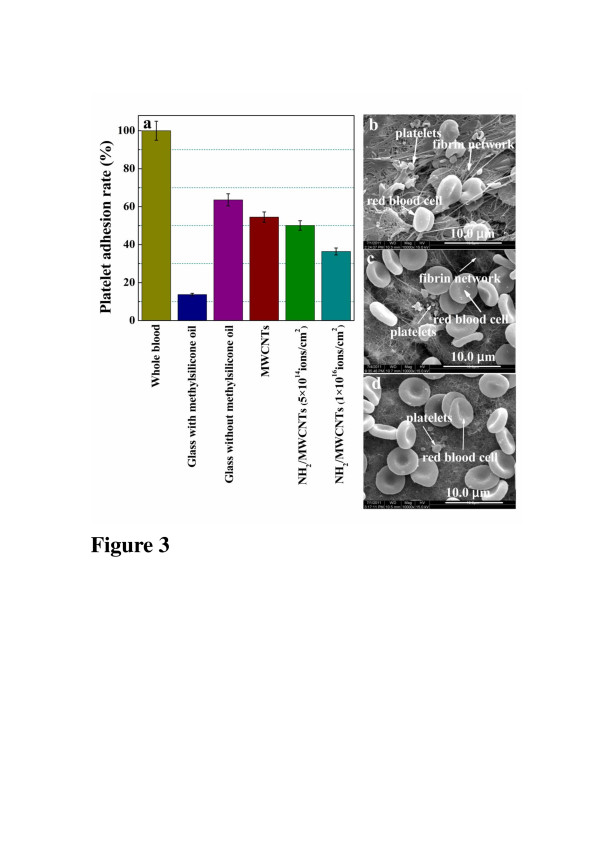
**Platelet adhesion rates of the samples and SEM images of RBCs and platelets.** (**a**) Platelet adhesion rates on different samples. SEM images of RBCs and platelets on (**b**) pristine MWCNTs, (**c**) NH_2_/MWCNTs with 5 × 10^14^ ions/cm^2^, and (**d**) NH_2_/MWCNTs with 1 × 10^16^ ions/cm^2^.

Platelet adhesion test is one of the simple and preliminary approaches to evaluate the hemocompatibility of biomaterials. Good surface antithrombogenicity is indicated by a small quantity of the platelets adhered on the surface, less activation, and morphological change. Figure 3a gives the platelet adhesion rates of different materials including the blank and the negative and positive control groups. It is clear that pristine MWCNTs and NH_2_/MWCNTs have lower platelet adhesion rate than the positive control group, interestingly that NH_2_/MWCNTs with 1 × 10^16^ ions/cm^2^ reveal the lowest platelet adhesion rate among all groups. The platelets which adhered on pristine MWCNTs and NH_2_/MWCNTs are observed by SEM (Figure 3b,c,d). Some fibrin network containing randomly distributed platelets can be seen on the surface of pristine MWCNTs. At the same time, the serious deformation of RBCs occurs (Figure 3b). Conversely, there are few fibrin networks or platelet aggregations on NH_2_/MWCNTs after exposure to platelet-rich plasma, as shown in Figure 3c,d, indicating insignificant thrombosis on both surfaces. Platelet adhesion and activation are the inevitable results of the interaction between blood and materials. It also can be seen that the morphology of RBCs on NH_2_/MWCNTs is perfect round. This result suggests that NH_2_/MWCNTs have no evident toxic effects on the red blood cells, which support superior hemocompatibility of NH_2_/MWCNTs. The hydrophilic surface induced by N-containing functional groups should be a main reason for inhibiting RBCs adhesion and deformation on the surface. This observation is consistent with the trend observed in the hemolytic rate test.

**Figure 4:**
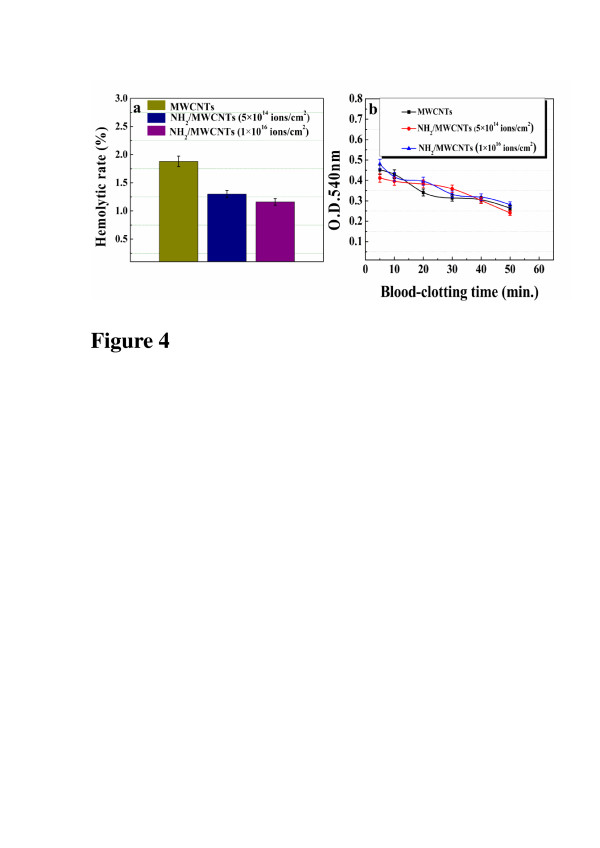
**Hemolytic rates and optical density values of MWCNTs and NH_2_/MWCNTs.** (**a**) Hemolytic rates of pristine MWCNTs and NH_2_/MWCNTs. (**b**) The OD_540 nm_ values of MWCNTs and NH_2_/MWCNTs vs. blood-clotting time.

Hemolysis is the loss of membrane integrity of RBCs leading to the leakage of hemoglobin into blood plasma [30]. It is one of the basic tests to understand the interaction of nanoparticles with RBCs. Nanoparticles might affect the membrane integrity of RBCs by mechanical damage or reactive oxygen species [31]. In addition, the hemolytic rate of nanoparticles can also be affected by their size, shape, surface charge, and chemical composition [32]. Figure 4a shows that, compared to pristine MWCNTs in which hemolytic rate is about 1.88%, NH_2_/MWCNTs display lower hemolytic rate, especially NH_2_/MWCNTs with fluency of 1 × 10^16^ ions/cm^2^.

The OD is used to evaluate the level of hemolyzed hemoglobin released from unclotted blood after contacting with the samples’ surface. Higher OD illustrates better thromboresistance. Figure 4b shows the OD of all samples at different blood-clotting times. Generally speaking, the blood starts to clot at 0.1 point of OD_540nm_ value at which the starting point of the kinetic blood-clotting time on the sample surfaces is recoded. It is clear that the kinetic blood time of all samples is longer than 50 min, revealing good hemocompatibility. The higher the OD is, the better thromboresistance. The OD of NH_2_/MWCNTs with 1 × 10^16^ ions/cm^2^ is a little bit higher than that of the other samples. Therefore, higher fluency of NH_2_^+^ implantation is related to better thromboresistance.

## Conclusions

Although MWCNTs has been widely investigated for diverse biomedical applications ranging from imaging and drug delivery to photothermal cancer ablation, strong thrombogenecity associated with this material as well as its propensities to induce hemolysis can potentially prohibit its applications. This work proves that NH_2_/MWCNTs are not endowed with any prothrombotic or platelet-stimulating characteristics nor do these compromise the integrity of the RBCs. In view of its significant properties, NH_2_/MWCNTs are expected MWCNTs derivative with potential for biomedical applications due to their lack of thrombotic and hemolytic predisposition.

## Abbreviations

CVD: Chemical vapor deposition; MWCNTs: Multiwalled carbon nanotubes; NH2/MWCNTs: NH_2_^+^ ion-implanted multiwalled carbon nanotubes; NS: Normal saline; OD: Optical density; RBCs: Red blood cells.

## Competing interests

The authors declare that they have no competing interests.

## Authors’ contributions

LDJ, SXL, DXY, and GHQ designed this work. GMX, ZML, and ZYT performed hemocompatibility experiments and observations. GMX, GDS, and LRY performed XPS, FTIR, SEM, and TEM measurements. GMX collected and analyzed data and wrote the manuscript. GHQ and WRX supported blood experiments. LDJ, SXL, and LRY revised the manuscript. All authors read and approved the final manuscript.
